# The outer membrane proteins based seroprevalence strategy for *Brucella ovis* natural infection in sheep

**DOI:** 10.3389/fcimb.2023.1189368

**Published:** 2023-06-14

**Authors:** Tao Zhang, Yu Wang, Yin Li, Tingting Qi, Zhirong Yue, Lili Cao, Bo Zhou, Huping Jiao

**Affiliations:** ^1^ College of Animal Science, Jilin University, Changchun, China; ^2^ Institute of Zoonosis, Jilin Academy of Animal Husbandry and Veterinary Medicine, Changchun, China; ^3^ Key Laboratory of Jilin Province for Zoonosis Prevention and Control, Institute of Military Veterinary, The Academy of Military Medical Sciences, Changchun, China

**Keywords:** natural infection, *Brucella ovis*, outer membrane proteins, epidemiological survey, sheep

## Abstract

**Introduction:**

The diagnosis of brucellosis largely relies on tiger red plate agglutination test (RBPT). However, it is difficult to distinguish between natural infection antibody positive and vaccination antibody positive, nevertheless, the identification of specific Brucella species natural infection.

**Methods:**

Here, we analyzed the structure of main outer membrane proteins (OMPs), OMP25 and OMP31 from *Brucella ovis* (*B. ovis*) and *Brucella melitensis* (*B. melitensis*), which are the main pathogens of sheep brucellosis, and found the OMP25 and OMP31 could be used as the differential antigens for *B. ovis* and *B. melitensis* antibody. Then we expressed the OMP25 from *B. ovis* (OMP25o) and OMP31 from *B. melitensis* (OMP31m).

**Results:**

They have equally efficiency in antibody detection of vaccinated sheep serum, consistent with the RBPT results. However, through epidemiological investigations, we found some RBPT positive samples were negative by the OMP31m based serum antibody detection, but these samples gave positive results by the OMP25o. We verified these OMP31m negative but OMP25o positive samples by *B. ovis* and *B. melitensis* specific primers based PCR detection, and all these samples were *B. melitensis* negative. However, four out of six samples are *B. ovis* positive. These results showed that we could use the OMP25o and OMP31m to diagnose sheep brucellosis antibody, especially to discriminate the infection of the *B. ovis*.

**Discussion:**

Currently, China has not yet approved a vaccine based on *B. ovis* and *B. ovis* positive samples should be naturally infected. There should be some implicit transmission of *B. ovis* in Jilin province. Further epidemiological investigation should be conducted to monitor the *B. ovis* natural infection.

## 1 Introduction

Brucellosis is caused by Gram-negative coccobacilli and facultative intracellular bacteria of the genus Brucella which contained several species according to their primary hosts: *B. melitensis* (goats), *B. abotus* (cattle), *B. suis (*pig), *B. ovis*(sheep), *B. neotomae*(wood rat) and *B. canis* (dogs) (Corbel, 1997). Brucella can colonize and proliferate for a long time after invading the host cell, form brucella-containing vacuoles to evade host immune response and induce persistent infection, which are difficult problems in the treatment and prevention of Brucellosis (Jiao et al., 2021). Brucellosis infection causes abortion in sheep and thus it brings serious economic losses to the sheep industry (Sun and Zhang, 2014). *B. ovis* is one of the main causative agents of sheep reproductive failure, with symptoms including chronic epididymitis, orchitis and infertility in sexually mature rams and occasional abortion and stillbirth in ewes (Ficapal et al., 1998). Once infected, transmission occurs between rams in direct contact or between rams that mate with the same ewe during the same breeding season (Ridler and West, 2011). Most rams continue to shed *B. ovis* in semen for at least 2 to 4 years (Ridler et al., 2006). In China, the sheep Brucella EPI vaccine is *B. melitensis* related species, which is believed it can also give the sheep protection against *B. ovis* infection (Schurig et al., 2002). The immunization of *B. melitensis* related vaccine gives rise the problem of discrimination between natural infection and immunization induced antibody detection. There is no suitable way for discrimination of natural infection and vaccination stimulated antibody, let alone the discrimination of natural infection of specific species.

Bacterial isolation and PCR based diagnosis method is the gold standard for the diagnosis of brucellosis (Saini et al., 2017), however, it is very time-consuming and difficult. Although PCR (Polymerase Chain Reaction) is the most sensitive detection strategy, the isolation of nucleic acids is difficult (Bricker, 2002; Lopez-Goni et al., 2008). The conventional testing methods of Brucella infection are based on agglutination test, such as plate agglutination test (PAT), tiger red plate agglutination test (RBPT), standard test tube agglutination test (SAT). These agglutination tests are based on the reactivity of antibodies against smooth lipopolysaccharide (LPS). Six typical species of Brucella, even some Gram-negative bacteria possess the lipopolysaccharide (Sotolongo-Rodriguez et al., 2022), which is known to be involved in the cross-reactions with closely related bacteria (Bulashev et al., 2020). So, it is impossible to discriminate the natural infection of Brucella and vaccination stimulated antibody in the vaccinated livestock farm based on the agglutination test.

Similar to other Gram-negative bacteria, Brucella cell also has two-layer membranes, the outer membrane and the inner membrane. The periplasm is in the two layers. The outer membrane can be sequentially extracted with different detergents. Three groups of proteins can be separated in their native state: designated as group 1 (90 KDa), group 2 (40 KDa) and group 3 (30 KDa) proteins according to their apparent molecular mass (Verstreate et al., 1982). Two proteins are identified from the group 3 proteins, the outer membrane protein OMP25 and OMP31, and they share 34% identity in amino acid sequence (Cloeckaert et al., 2002). The apparent molecular mass of OMP25 and OMP31 is 25-34 kDa. OMP31 appears as immunodominant antigen in the course of *B. ovis* infection (Estein et al., 2003). Although, the gene of *omp31* is deleted in *B. abortus* (Vizcaino et al., 2001), the vaccination of recombinant OMP31 in mice can confer protection against *B. ovis* and *B. melitensis* infection (Cassataro et al., 2005). OMP31 also can be used as the antigen for serological diagnosis of Brucella infection (Gupta et al., 2007). OMP25 was firstly identified from *B. abortus* (Cloeckaert et al., 1996b). Amino acid analysis shows that OMP25 displays about 40% identity with other *Rhizobiaceae* group members, such as *Agrobacterium tumefaciens*. Different from OMP31, OMP25 is highly conservative in Brucella species, which is the main virulence factor of Brucella (Cloeckaert et al., 1996a). OMP25 involves in the inhibition of macrophage TNF-α production, which is the first cytokine produced in Brucella infection (Jubier-Maurin et al., 2001). The DNA sequence variation is less than 1.9% among different Brucella species. Interestingly, the *omp25* DNA sequence of *B. ovis* has a short deletion of 36nt at the 3′ end of *omp*25 gene. This deletion causes an antigenic shift in both linear epitopes and non-linear epitopes (Cloeckaert et al., 1996a).

Through structural analysis of OMP25 and OMP31 of *B. ovis* and *B. melitensis*, we speculate that sheep should generate some special antibody repertoires against *B. ovis* OMP25(OMP25o) different from *B. ovis* OMP31, which is almost similar with *B. melitensis* OMP31(OMP31m). This property can be used for discrimination of *B. melitensis* antibody (including vaccine stimulated antibody) and *B. ovis* antibody. In China, there is no *B. ovis* based vaccine, the *B. ovis* related antibody positive individuals should be the result of natural infection with *B.ovis*. This strategy can at least be used in screening for the *B. ovis* natural infection, providing guidance for farmers to handle their breeding sheep.

## 2 Materials and methods

### 2.1 Bacterial strains

The DH5α, BL21 competent cells are purchased from TIANGEN BIOTECH (https://www.tiangen.com).

Structural analysis of OMP25 and OMP31 is based on the Alfa fold online instruction (https://colab.research.google.com/github/sokrypton/ColabFold/blob/main/AlphaFold2.ipynb).

### 2.2 Recombinant Brucella OMPs

The sequence of omp25 gene (642 bp) was download from Genebank (AAB06702.1). It was cloned into the pET-28a expression vector and designated as pET-28a-omp25. The sequence of omp31 gene (723 bp) was download from Genebank (AAB36693.1). It was cloned into the pET-28a expression vector and designated as pET-28a-omp31. Recombinant OMP25o and OMP31m were expressed as inclusion bodies in Escherichia coli (BL21) by induction with 1.25 mM isopropyl thiogalactoside (IPTG). OMP25o and OMP31m extracts were denatured by 8 M urea and purified by Ni-NTA agarose (Sangon Biotech, www.sangon.com), and then refolded by dialysis against 20 mM Tris-HCl buffer containing 0.1 mM DTT with β-ME, 1 mM reduced glutathione and 0.2 mM oxidized glutathione. Aliquots were run on SDS to check the recombinant protein OMP25o and OMP31m. Purified soluble OMP25o and OMP31m were used for serological tests.

Before proceeding with a large-scale preparation, we optimized the induction conditions, including the concentration of IPTG and the induction time. The results are shown in **Figures S1**, **S2**. The modified pET-28a+ expression plasmid encoding omp25 or omp31 and fusion proteins were individually introduced into BL21 (DE3) competent cells (TIANGEN). After overnight incubation at 37°C on Luria-Bertani (LB) agar plates supplemented with 50 μg/mL kanamycin, a single positive colony was inoculated into 10 mL of LB medium with 50 μg/mL kanamycin and then grown at 37°C overnight with shaking at 220 rpm. The resulting seed culture was added to a 1 L baffled conical shake flask containing 500 mL of LB medium with 50 μg/mL kanamycin. Incubation at 37°C with shaking at 220 rpm until culture absorbance (600 nm) of 0.6 was reached. IPTG was added to a final concentration of 1.25 mM for induction at 37°C for 8 h or 6 h with shaking at 220 rpm. The bacterial cultures were harvested by centrifugation at 8000g and 4°C for 15 min.

To purify OMP25o and OMP31m, cell pellets were resuspended in lysis buffer (20 mM PBS, pH 7.2-7.6, 8 M Urea, 5 mM imidazole) and lysed using TGyrate Basic Vortex mixer (TIANGEN). The cell debris was removed by centrifugation at 8000g for 30min. Filtered the supernatant using a Millipore cartridge (0.22 μm pore size) and loaded it onto a gravity flow column containing Ni-NTA resin. The target protein was eluted with elution buffer (20 mM PBS, pH 7.2-7.6, 8 M Urea, 500 mM imidazole). Peak fractions were analyzed by SDS-PAGE to determine whether the target protein was collected. After confirming the purity and yield of the protein, the fractions were pooled, concentrated, and stored at 4°C.

### 2.3 Blood and serum samples

All samples are sheep originated. The samples are detected by Rose Bengal test and 100 samples are identified as positive samples, 33 as negative samples. There are 100 samples that are not tested. (Samples come from various regions of Jilin province such as Shulan, Nong’an, Da’an, Tongyu, Dehui). All serum samples are treated with 60°C water bath for 30 min before use. The blood samples are freshly used to extract nucleic acids.

### 2.4 Western blot

OMP25o and OMP31m were electrophoresed on SDS-PAGE and transferred to Polyvinylidene Fluoride membranes (PVDF membranes, Millipore, Billerica, Massachusetts, USA). The blotted membrane was incubated in 1:1,000 diluted monoclonal antibody for 2 h at room temperature. The strip was washed in TBS containing 0.05% Tween 20 (TBST) and incubated with 1:5,000 dilution of goat anti-mouse IgG and IgM HRP-conjugated antibodies. The blot was visualized by adding immuno-chemiluminescence reagent (ECL, Millipore, Billerica, Massachusetts, USA).

### 2.5 ELISA with OMP25o and OMP31m

Coat the antigen onto the ELISA plates by adding 100 μl of the antigen solution to each well. Incubate overnight (about 12 h) at 4°C. Wash the wells three times with PBST (0.05% Tween 20 in PBS). Block the rest of the protein binding sites with 200 μl/well blocking buffer (3% BSA in PBS), and put the plates in 37°C oven for 2 h. After washing the plates three times with PBST, dilute the serums in an appropriate ratio with PBST containing 3% BSA and dispense it to the wells. Incubate at 37°C for 1 h. Wash the plates three times with PBST. Adding the donkey anti sheep IgG HRP-conjugated antibody solution (https://www.abcam.cn) to bind the specific antibodies in serums. Incubate for 40 min at 37°C. Wash the plates three times with PBST. Add chromogenic substrate TMB and after 10 min incubation has elapsed, stop the reaction with 2 M H_2_SO_4_. Finally, the optical densities at 450 nm wavelengths were measured on Microplate Reader.

Using Omp25 and Omp31 as coating antigens, the experiment was carried out according to the best experimental conditions of the pre-experiment.

### 2.6 DNA extraction and PCR verification

Bloods were collected using vacuum tubes treated with heparin and 0.5 ml of blood was used to extract DNA. The blood was centrifuged at 500g for 7 min and the supernatant discharged. The pellets were suspended in 1 ml red blood cell lysis buffer (155 mM NH_4_Cl, 12 mM NaHCO_3_, 0.1 mM EDTA) and incubated on ice for 4 to 5 min with occasional shaking. White blood cells were collected by centrifugation at 500g for 5 min and used for DNA extraction by silica column (MolPure^®^ Blood DNA Kit) according to manufacturer instructions. The PCR primer for *B. ovis* is previously validated species-specific PCR primer for *B. ovis* detection (Forward 5′-GCCTACGCTGAAACTTGCTTTTG-3′ and Reverse 5′-ATCCCCCCATCACCATAACCGAAG-3′), which corresponding to a 228 pb amplicon.

## 3 Results

### 3.1 Paired comparative analysis of the sequence and structure of OMP25 and OMP31 in *B. ovis* and *B. melitensis*

**Figure 1:**
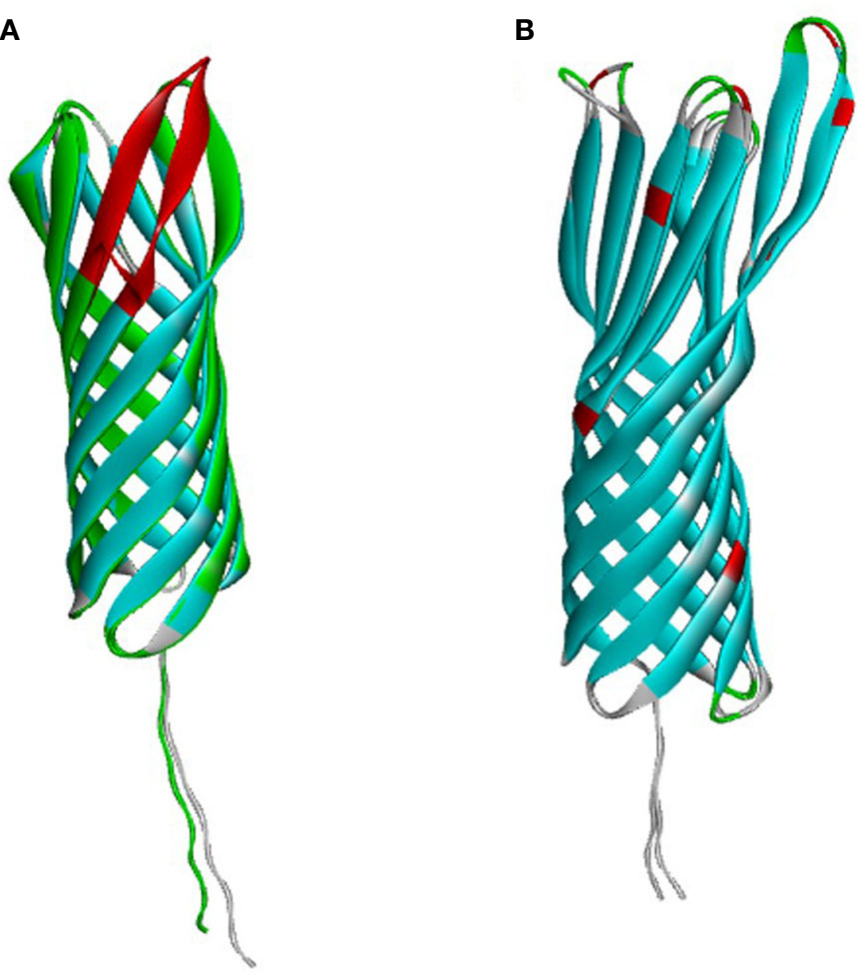
The Alfa-fold based structure of OMP25 and OMP31. **(A)** OMP25. **(B)** OMP31.

OMP25 and OMP31 are the major outer membrane proteins, which have dominant roles in the virulence and immune response of Brucella. Firstly, we compared the sequence and structure of OMP25 and OMP31 in *B. ovis* and *B. melitensis* separately. OMP31 of *B. melitensis* (OMP31m) is a 31 kDa pore-forming protein with 240 amino acid residues, having a β-barrel structure based on eight β-strands with flexible N-terminal of 48 residues, which is the linker of peptidoglycan (Godessart et al., 2021) (**Figure 1A**). OMP31 of *B. ovis* (OMP31o) also has 240 amino acids with 9 residues sporadically spread along the sequence. The identical Tm-score is 0.97. They are very similar to each other, which maybe the reason why immunity based on *B. melitensis* can also give protection against *B. ovis* infection. On the other hand, OMP25 of *B. melitensis* (OMP25m) is a 23 kDa protein consisting of 213 residues, having similar three-dimensional structure with OMP31 (**Figure 1B**). However, OMP25 of *B. ovis* (OMP25o) is short of 12 amino acid residues on the C terminal corresponding to the fourth loop of seventh and eighth strands. This could cause antigenic shift in both linear and non-linear epitopes, which could be used in the identification of *B. ovis* infection (Cloeckaert et al., 2002). So, we selected OMP31m and OMP25o as candidate antigens for Brucella infection detection.

### 3.2 Expression and Purification of OMP31m and OMP25o

**Figure 2:**
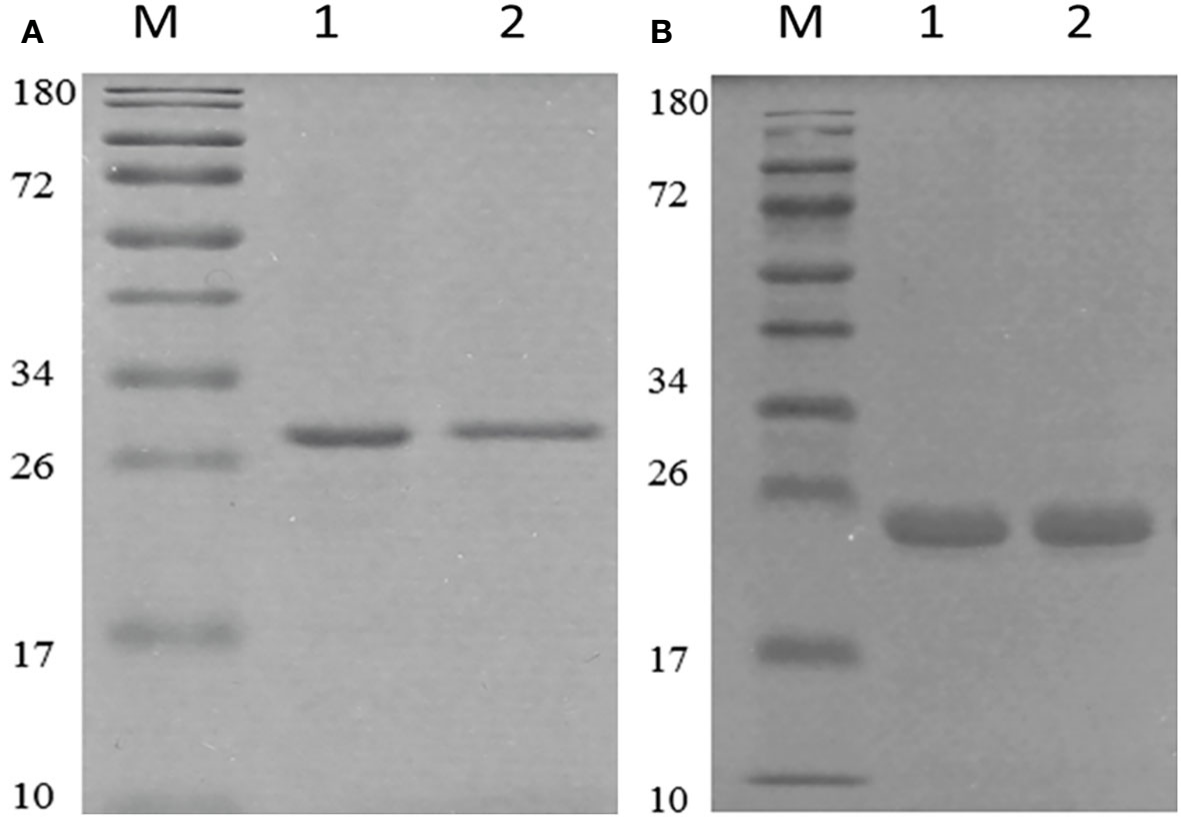
Purification and identification of recombinant OMP25o and OMP31m. **(A)** The purification of OMP25o. M, 10-180KDa protein marker; 1, purified protein; 2, refolded protein. **(B)** The purification of OMP31m M, 10-180KDa protein marker; 1, purified protein; 2, refolded protein.

We used the pET-28a+ as the expression vector, which has a lot of merits in protein expression and purification. After codon optimization, the correct vector was transferred into BL21(DE3) cells. The optimization of expression conditions showed that under the condition of 1.25 mM ITPG, the target protein could express very well as inclusion body (**Figure S3**). However, OMP25 had the highest expression with 8 hours of induction, while the corresponding time is 6 hours for OMP31. The proteins were purified using Ni-NTA column under denaturation condition. Renaturation of the target protein was performed using PBS dialysis method. The SDS-PAGE results showed that we obtained pure target proteins (**Figure 2**). Due to the denaturation and renaturation of obtained proteins, monoclonal antibodies corresponding to the target proteins were used in Western blot to prove their immunogenicity and reactivity. As shown in **Figure S4**, we could obviously observe positive results of the reaction at 25 and 31 KDa. The results indicated that our recombinant Omp25o and Omp31m have excellent immunogenicity and reactivity.

### 3.3 OMP31m and OMP25o based indirect ELISA is equivalent to RBPT in detecting vaccine immunized and non-immunized sheep serum

**Figure 3:**
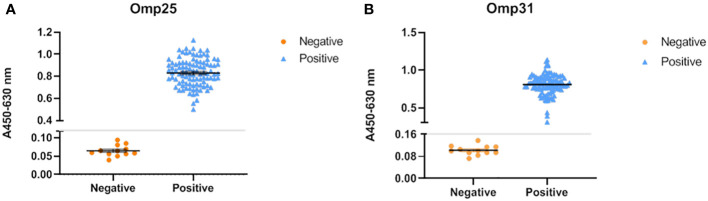
Reactivity of OMP25 and OMP31 with Brucella serum samples by ELISA. **(A)** The recombinant OMP25 protein was used as the antigen (5μg/mL) to coat the plate, and the serum was diluted at a ratio of 1:10; 100 positive serums and 32 negative serums, of which 20 negative serums were used to calculate the cut off. The test results are shown in the figure: orange represents the test result of negative serum, blue represents the result of positive serum, and the gray line is the cut off value. **(B)** The recombinant OMP31 protein was used as the antigen (10μg/mL) to coat the plate, and the serum was diluted at a ratio of 1:80; 100 positive serums and 32 negative serums, of which 20 negative serums were used to calculate the cut off. The test results are shown in the figure: orange represents the test result of negative serum, blue represents the result of positive serum, and the gray line is the cut off value.

To determine the ability of our recombinant proteins in serum screening, indirect ELISA was chosen with Omp25o and Omp31m as coating antigens. The optimal coating conditions were determined by checkerboard experiment. The results showed that the optimal coating concentration of Omp25o is 8 μg/ml and the optimal dilution ratio is1:10 (**Table S1**). The optimal coating concentration of Omp31m is 10 μg/ml and the optimal dilution ratio is 1:80 (**Table S2**). 100 known vaccine immunized serum samples and 33 known negative samples were chosen for indirect ELISA detection. The results showed that they were completely consistent with the results of the Rose Bengal test (**Figure 3**). It indicated that recombinant protein has excellent immunogenicity and can accurately identify serum through indirect ELISA.

### 3.4 OMP31m and OMP25o based indirect ELISA is different in epidemiological survey

**Figure 4:**
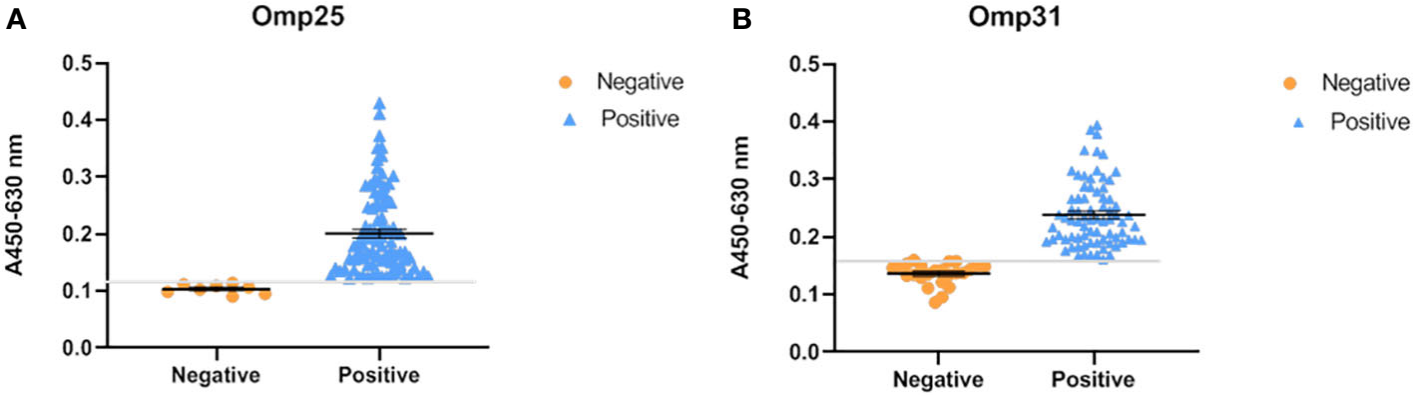
OMP25 and OMP31 based epidemiological survey. **(A)** Reactivity of OMP25 with Brucella serum samples by ELISA. Orange represents the test result of negative serum, blue represents the result of positive serum, and the gray line is the cut off value. **(B)** Reactivity of OMP31 with Brucella serum samples by ELISA. The test results are shown in the figure: orange represents the test result of negative serum, blue represents the result of positive serum, and the gray line is the cut off value.

To evaluate the prevalence of brucellosis infection in these areas (as mentioned above), we randomly selected 100 sheep serum samples and tested them using our established indirect ELISA method. According to the cut off value, the results showed that the positive detection rate is 82/100 when the antigen is OMP25o (**Figure 4A**), and the positive detection rate is 76/100 when the antigen is OMP31m (**Figure 4B**). Since these sheep have already been vaccinated against brucellosis, our test results indicated that the antibodies produced by their immune system were still at a certain level. Moreover, three replicates were performed on each sample, and the results showed favorable repeatability. Notably, six samples are only OMP25o positive, and the positive value is absolutely higher than the cut off value. Therefore, we retested theses six samples with RBPT and the results were also positive. That means that we found six samples with only OMP25o positive results. RBPT detection is based on Brucella extracts complex antigen, indicating that the sample is Brucella positive. However, these samples were *B. melitensis* negative, but *B. ovis* positive. So, we decided to verify the sample by nucleic acids PCR test.

### 3.5 PCR verification of candidate *B. ovis* positive samples

**Figure 5:**
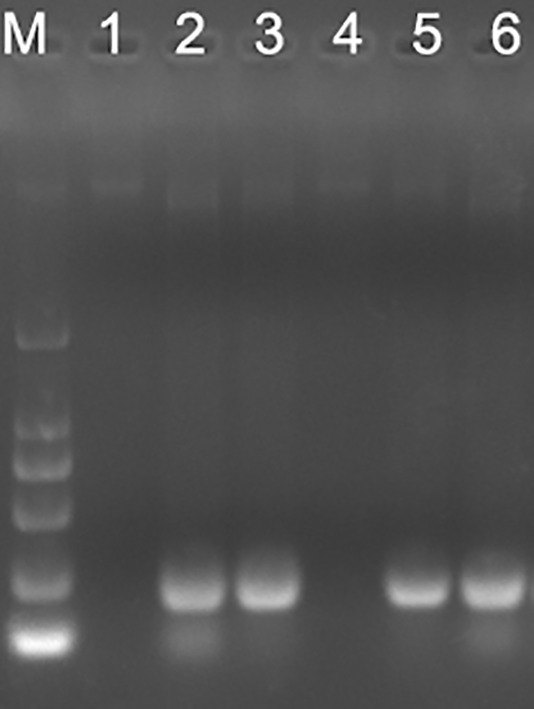
PCR verification of the *omp25* positive and *omp31* negative samples. M: DL2000; 1: sample 1; 2: sample 2; 3: sample 3; 4 sample 4; 5: sample 5; 6: sample 6.

PCR is the most sensitive method for causative agent detection. So, blood DNA was extracted and purified from OMP25o positive and OMP31m negative samples. *B.ovis* specific primers were designed and synthesized according to previous research results (Ilhan et al., 2008). The PCR product has a total length of approximately 228bp. Four out of six only OMP25o positive samples could get positive results through *B.ovis* specific primers PCR (**Figure 5**). It is acceptable that we cannot verify all *B. ovis* antibody positive samples with PCR, because this bacterium is not always detected in the blood. This result indicated that there might be undetected *B. ovis* natural infection in Jilin Province. Our indirect ELISA strategy based on OMP31m and OMP25o recombinant antigens could easily screen for natural infection of *B. ovis.* Epidemiological survey should be conducted in Jilin province on *B. ovis* natural infection.

## 4 Discussion

*B. ovis* is one of the main pathogens of sheep brucellosis. Since it can produce subclinical asymptomatic infections, it will have long-term latent transmission in sheep flocks and cause serious economic losses to sheep industry. So, the diagnosis of *B. ovis* infection is very important for the clearance of *B. ovis* infection in sheep flock. However, there is currently no suitable strategy to screen for natural infections of *B. ovis.* We first compared the structural characteristics of OMP25 and OMP31, all of them are barrel shaped proteins. OMP31m and OMP31o are highly similar, with only 9 amino acids substituted. However, OMP25o lacks 12 amino acids in the carbon terminal, which corresponds to the fourth loop of the beta barrel protein, means that OMP25o will produce some special antibodies completely different from that of OMP25m. Therefore, OMP25o and OMP31m were selected as candidate antigens to distinguish *B. ovis* induced antibody from *B. melitensis* induced antibody. Since OMP25 and OMP31 are beta barrel structure, their expression takes the form of inclusion body. After purification and renaturation of our target proteins, we first tested their ability to detect serum antibodies after inoculation. We found that these two proteins were equal in detection of vaccinated serum antibodies and the results were consistent with traditional RBPT method. However, when we applied these two proteins in epidemiological surveys, the results showed that OMP25o was more sensitive than OMP31m, and the result of OMP25o was consistent with that of RBPT. So, we reasoned that our epidemiological samples should have *B. ovis* antibody positive samples. PCR confirmed that our samples indeed contained *B. ovis.* Four out of six samples tested positive. Our indirect ELISA based on two antigens is an effective serum screening strategy that can overcome the drawback of false negative in PCR. If this strategy can combine with PCR detection, the diagnosis of brucellosis will be more accurate. It will be realizable to find a sheep flock without brucellosis.

## Data availability statement

The original contributions presented in the study are included in the article/**Supplementary Material**. Further inquiries can be directed to the corresponding authors.

## Ethics statement

The animal study was reviewed and approved by Institutional Animal Care and Use Committee of Jilin University. Written informed consent was obtained from the owners for the participation of their animals in this study.

## Author contributions

TZ and YW conduct the experiment. TQ, YL, and ZY calculate and statistic the data. BZ and HJ designed the experiment and written the manuscript. LC analyzed data and interpretation. All authors contributed to the article and approved the submitted version.
