# Supplementary material for: The outer membrane proteins based seroprevalence strategy for Brucella ovis natural infection in sheep
Source: Front Cell Infect Microbiol. 2023 Jun 14;13:1189368. doi: 10.3389/fcimb.2023.1189368 (PMC10302724; doi:10.3389/fcimb.2023.1189368)
Supplement: Supplementary file 1 [file DataSheet_1.docx]

Supplementary Material

The outer membrane proteins based seroprevalence strategy for *Brucella ovis* nature infection in sheep

Tao Zhang, Yu Wang, Yin Li, Tingting Qi, Zhirong Yue, Lili Cao, Bo Zhou, Huping Jiao

*** Correspondence:** Huping Jiao:[jiaohp@jlu.edu.cn](mailto:jiaohp@jlu.edu.cn)

# Supplementary Figures and Tables

## Supplementary Figures


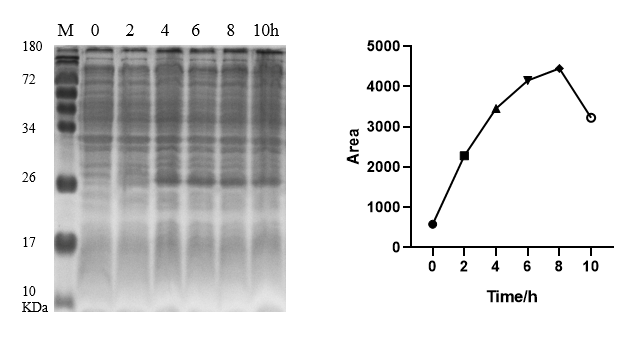

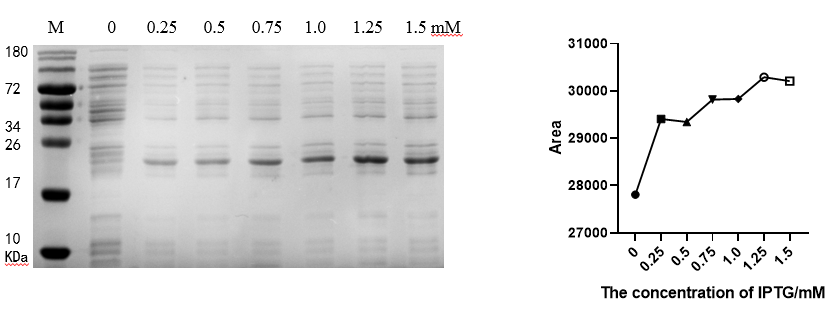

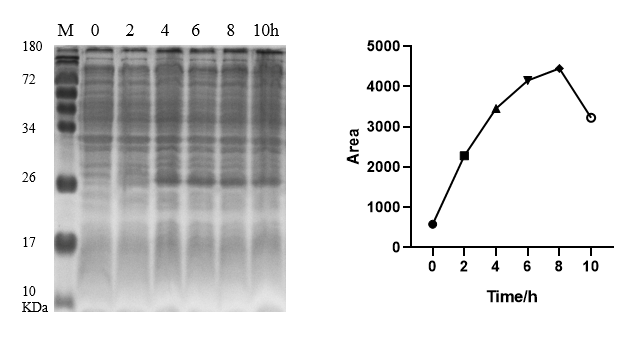

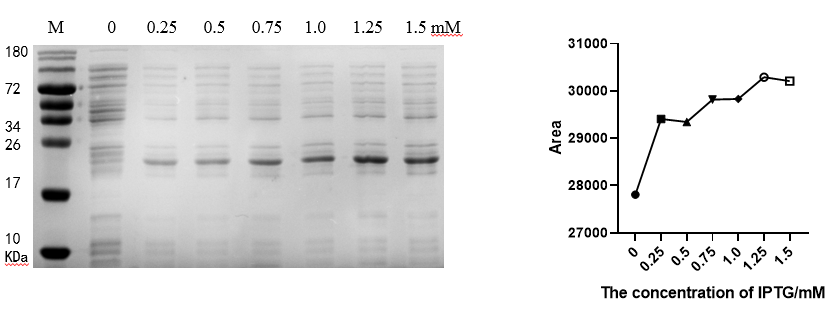


**Supplementary Figure 1.** Optimization of OMP 25 expression conditions


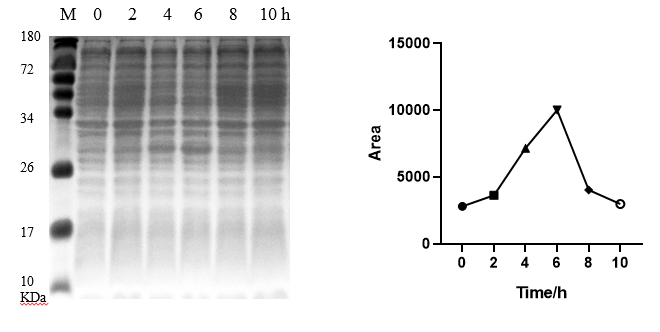

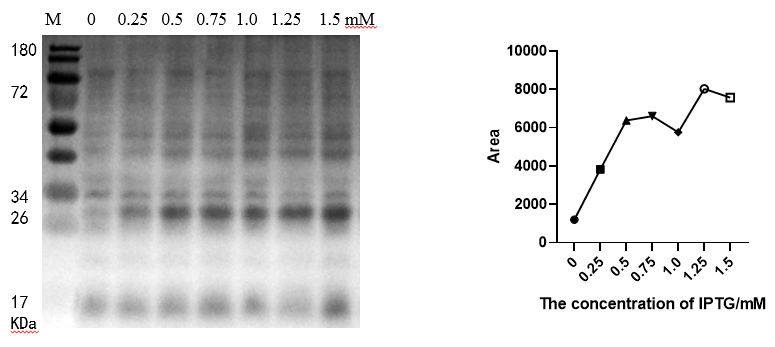

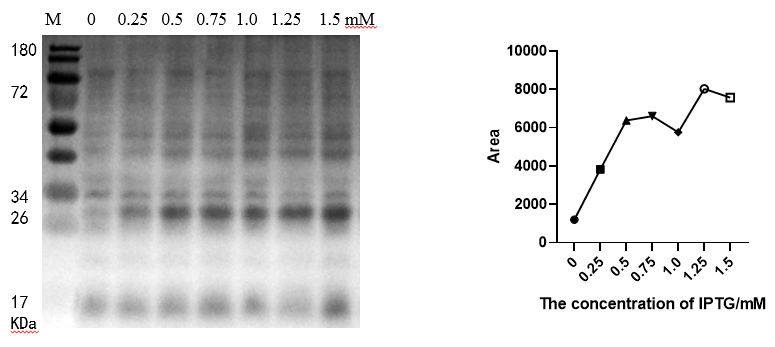


**Supplementary Figure 2.** Optimization of OMP 31 expression conditions


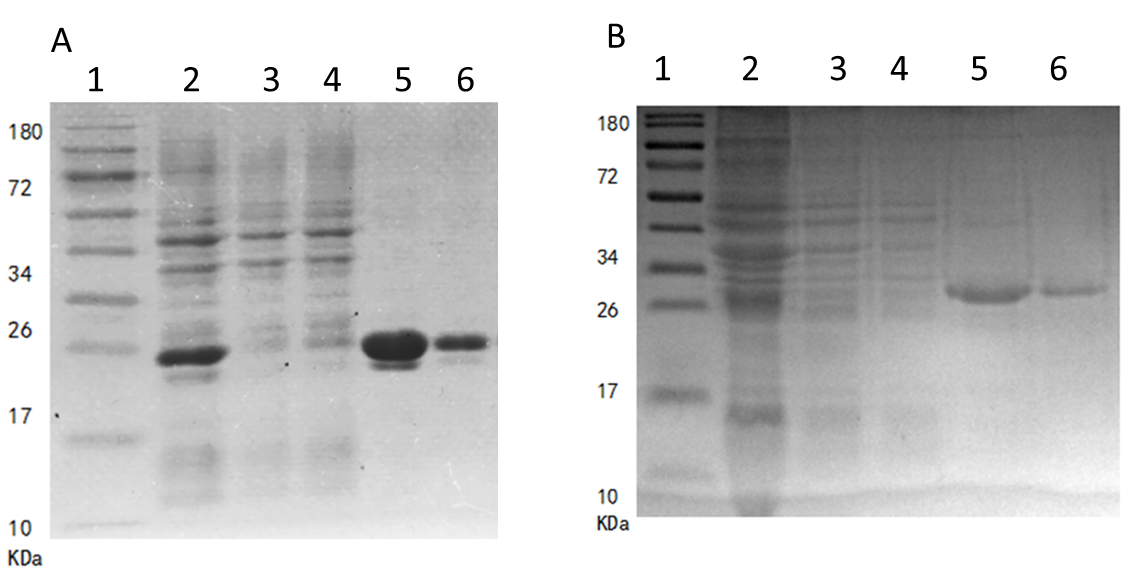


**Supplementary Figure 3.** Purification and identification of recombinant Omp25 and Omp31 **(A)** The purified rOmp25 was analyzed by SDS-PAGE. Lane 1, 10-180KDa marker; Lane 2, cell lysate; Lane 3,flow through; Lane 4,impurified protein eluted by 5mM Imidazole ;Lane 5,6 purified protein eluted by 500mM Imidazole. **(B)** The purified rOmp31 was analyzed by SDS-PAGE. The purified rOmp31 was analyzed by SDS-PAGE. The description of lane is the same as above.


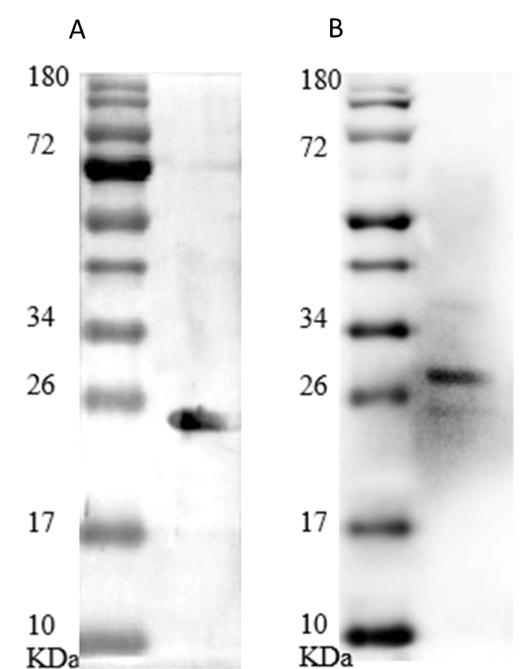


**Supplementary Figure 4.** Reactivity of mAb with OMPs **(A)** Reactivity of mAb with the denatured rOMP25 (2st panel)in WB. The first panel showed the 10-180 marker by WB. **(B)** Reactivity of mAb with the denatured rOMP31 (2st panel) in WB. The first panel showed the 10-180 marker by WB.

## Supplementary Tables

**Supplementary Table 1** The checkerboard titration of OMP 25

| Serum Dilution | OMP 25 （μg/mL）Antigen Dilution | | | | | | |
| --- | --- | --- | --- | --- | --- | --- | --- |
|  | 10 | 8 | 6 | 4 | 2 | 1 | 0.5 |
| 1:10 | 0.837 | 0.867* | 0.813 | 0.757 | 0.689 | 0.577 | 0.481 |
| 1:20 | 0.706 | 0.652 | 0.648 | 0.596 | 0.453 | 0.464 | 0.399 |
| 1:40 | 0.717 | 0.649 | 0.612 | 0.540 | 0.397 | 0.353 | 0.331 |
| 1:80 | 0.650 | 0.611 | 0.520 | 0.471 | 0.348 | 0.260 | 0.232 |
| 1:160 | 0.561 | 0.535 | 0.457 | 0.357 | 0.231 | 0.171 | 0.136 |
| 1:320 | 0.407 | 0.396 | 0.349 | 0.263 | 0.162 | 0.107 | 0.092 |
| 1:640 | 0.333 | 0.273 | 0.235 | 0.171 | 0.098 | 0.066 | 0.043 |
| 1:1280 | 0.249 | 0.183 | 0.154 | 0.124 | 0.070 | 0.044 | 0.030 |

The best coating concentration of OMP 25: 8μg/mL, the best dilution ratio: 1:10.

**Supplementary Table 2** The checkerboard titration of OMP 31

| Serum Dilution | OMP 31 （μg/mL）Antigen Dilution | | | | | | |
| --- | --- | --- | --- | --- | --- | --- | --- |
|  | 10 | 8 | 6 | 4 | 2 | 1 | 0.5 |
| 1:10 | 0.026 | 0.650 | 0.677 | 0.791 | 0.655 | 0.502 | 0.481 |
| 1:20 | 0.766 | 0.804 | 0.868 | 0.706 | 0.585 | 0.482 | 0.450 |
| 1:40 | 0.768 | 0.862 | 0.764 | 0.622 | 0.539 | 0.458 | 0.421 |
| 1:80 | 0.923* | 0.781 | 0.738 | 0.644 | 0.456 | 0.373 | 0.341 |
| 1:160 | 0.825 | 0.728 | 0.612 | 0.482 | 0.339 | 0.274 | 0.253 |
| 1:320 | 0.696 | 0.606 | 0.492 | 0.401 | 0.261 | 0.212 | 0.177 |
| 1:640 | 0.551 | 0.462 | 0.371 | 0.255 | 0.163 | 0.130 | 0.110 |
| 1:1280 | 0.414 | 0.352 | 0.229 | 0.178 | 0.110 | 0.072 | 0.062 |

The best coating concentration of OMP 31: 10μg/mL, the best dilution ratio: 1:80.
